# The infectivity of AAV9 is influenced by the specific location and extent of chemically modified capsid residues

**DOI:** 10.1186/s13036-024-00430-7

**Published:** 2024-05-14

**Authors:** Sergio Milagros, Pablo Ramírez-Ruiz de Erenchun, Maite Guembe, Beatriz Carte, Miriam Méndez, Ander Uribarri, Rafael Aldabe

**Affiliations:** DNA and RNA Medicine Division, CIMA Universidad de Navarra, 31008 Pamplona, Spain

## Abstract

**Background:**

Several treatments for genetic diseases utilizing recombinant adeno-associated viruses (AAVs) have recently gained approval. However, the development of a greater number of therapeutic AAVs is constrained by certain limitations. While extensive efforts have concentrated on screening AAV genetic libraries, an alternative strategy involves modifying the AAV capsid by attaching various moieties. The capsid of AAV plays a pivotal role in transducing target cells and evading immune responses, making modifications a key avenue for engineering improved variants.

**Results:**

In our study, we replaced specific AAV9 capsid residues with an unnatural amino acid bearing a bioorthogonal group, identifying four positions with no adverse impact on production. Utilizing click chemistry, we attached varying proportions of Cy5.5 to these positions, allowing us to assess the impact of these modifications on AAV9 infectivity in cultured cells. Our findings reveal that both the position and degree of capsid modification significantly affect AAV transduction. While higher amounts of attached molecules lead to an increased number of AAV genomes within cells, this does not positively impact transgene expression. Conversely, a negative impact on transgene expression is observed when the AAV capsid is highly modified, with the degree of this effect associated with the modified residue.

**Conclusion:**

Careful control of both the degree and specific position of capsid modifications is crucial for optimizing transduction efficiency and minimizing undesired effects on transgene expression. These results underscore the importance of precision in AAV capsid modification to achieve optimal transduction efficiency while mitigating potential drawbacks on transgene expression.

**Supplementary Information:**

The online version contains supplementary material available at 10.1186/s13036-024-00430-7.

## Background

Gene therapy based on recombinant adeno-associated virus (rAAV) has revolutionized the clinical management of various genetic diseases, with approved AAV drugs either by the EMA, the FDA or both: Glybera [1] (lipoprotein lipase deficiency), Luxturna [2] (for Leber congenital amaurosis), Zolgensma [3] (for spinal muscular atrophy), Roctavian [4] (for Hemophilia B), Upstaza [5] (for severe aromatic L-amino acid decarboxylase deficiency) and Hemgenix [6] (for Hemophilia B). Over a hundred clinical trials using AAV drugs have been conducted to correct defective human genes, establishing AAV as the leading in vivo gene delivery system due to its low immunogenicity, customizable design, and safety profile. Despite its rapid advancements, certain challenges remain, including limited tissue tropism, suboptimal transduction efficacy in specific tissues, pre-existing neutralizing antibodies (NAbs), and the requirement for high vector dosages [7, 8]. Specifically, optimizing target-specific vectors, enhancing transduction efficiency, and ensuring immune evasion are key areas that require further advancement. Overcoming these hurdles will be instrumental in expanding the impact and effectiveness of AAV-based therapies in diverse clinical settings.

The target tissues and cell types for transgene expression are primarily determined by AAV capsid, and different wild-type AAV serotypes exhibit distinct tissue tropisms due to variable regions interacting with specific cell surface receptors [9, 10]. Therefore, the AAV capsid must engage with various host organism components to facilitate effective transduction of target cells. The binding of AAVs to cell surface glycans is believed to act as attaching factors that enhance internalization by increasing interactions with transmembrane receptor proteins responsible for entry. The presence of specific types of glycans can influence binding and internalization in a manner specific to each serotype, potentially affecting tropism. Heparan sulfate proteoglycan was identified as an essential component for cell binding and transduction for AAV2, establishing it as the first recognized receptor [11]. Subsequent research revealed specific glycans for other serotypes, such as sialic acid moieties for AAV5, -1, -6, and -4, and galactose for AAV9 [12–15]. Glycans are accepted as 'primary receptors' implying an early interaction, characterized by low-affinity, indiscriminate binding of these glycans, which lead to sequestration and concentration of AAV at the cell plasma membrane. Distinct glycan affinities could play a pivotal role in determining tissue and host compatibility. However, the specificity provided by the glycans seems relatively limited.

In 2016 AAV receptor (AAVR) was recognized as an essential receptor for the entry of various AAV serotypes [16]. While AAVR was initially the main focus, it's evident that more than 40 host genes influence AAV transduction acting as partners or engaging with AAV at different stages along an AAVR-mediated pathway playing a role in entry, trafficking, and uncoating processes. Recently GPR108 has been described as an AAV entry factor, predominantly found in the Golgi, suggesting its involvement in virus trafficking or escape mechanisms [17, 18]. Moreover, ATP2C1, that encodes a membrane calcium transport pump, is crucial for upholding cellular calcium balance and impacts on AAV transduction revealing that proper cellular calcium levels are necessary for effective intracellular trafficking and structural alterations in the AAV capsid, as it has been observed in other viruses [19]. These factors all adhere to specific locations on the capsid to function as transduction agents. Furthermore, while glycan-binding sites across different serotypes are typically situated similarly, they do not exhibit amino acid alignment in phylogenetic comparisons.

Consequently, AAV tropism remains broad and unspecific, limiting their application in certain tissues of interest. Additionally, pre-existing NAbs in the human population against specific AAV serotypes can impede effective transduction or render patients ineligible for treatment. To overcome these limitations, researchers worldwide have explored modifications to the AAV capsid to expand its potential applications. Various approaches have been investigated, ranging from genetic engineering to chemical modifications, in the development of newly engineered capsids [20]. Modified versions of AAVs encompass a spectrum of approaches, from genetic modifications of the viral cap gene to conjugation of proteins like streptavidin or antibodies to enhance interactions with specific cells. Previous studies have demonstrated that incorporating distinct genetic sequences into the VP2 gene can influence AAV9's targeting to various cells within bone tissue [8] and AAV6's transduction efficiency in diverse cancer cells [8]. For instance, Lee and Ahn found that AAV2 modified with a streptavidin-biotin complex linked to an anti-EpCAM antibody exhibited significant efficacy in targeting EpCAM-positive ovarian cancer cells [21].

Numerous research teams have utilized DNA shuffling and peptide display methods to create innovative AAV capsids with enhanced in vivo transduction capabilities. These novel AAV vectors have shown success in targeting various organs, such as the liver, brain, skeletal muscle, and eye [20]. Additionally, recent efforts have focused on synthetically modifying AAV capsids to improve properties like immune evasion and cell-specific receptor targeting. The presence of glycan binding sites of different serotypes in similar general locations, despite they do not share amino acids that align phylogenetically gave hope for retargeting gene therapy vectors. Consequently, modifications in heparin-binding and the integration of an AAV9-like galactose binding site into AAV2 have resulted in alterations of transduction characteristics, ultimately leading to improved transduction efficiency [22].

Ultimately, chemical modifications of the AAV capsid have unveiled novel pathways for enhancing the properties of recombinant AAVs. Introducing GalNac or Mannose to exposed tyrosines has notably enhanced transgene expression in the liver and retina [23]. Moreover, the impact can be serotype-dependent; for instance, binding N-ethyl Maleimide to the AAV9 capsid has augmented its affinity for murine bone marrow while diminishing transduction in liver tissue, unlike AAV2 and AAV8 which did not exhibit this effect [24]. However it is challenging to generate modifications highly specific and selective, targeting only the desired regions of the capsid.

Emerging developments in synthetic biology have opened doors to the incorporation of a reactive unnatural amino acid onto the surface of the AAV capsid, allowing for precise click chemistry conjugation with molecules at designated sites. For this purpose, sets of distinct prokaryotic tRNA/tRNA synthase pairs have been designed and introduced into mammalian cells, enabling the integration of unnatural amino acids into newly forming polypeptides [25]. This approach has been applied in AAV engineering, introducing an azido group onto the AAV capsid surface to facilitate click chemistry conjugation with molecules such as oligonucleotides [26], aptamers [27], and various chemicals like PEG and folic acid [27, 28]. However, modifications using azido-lysine at different positions across different AAV serotypes (AAV2, AAV8, AAV-DJ/8 and AAVLK03) have shown restricted transduction efficiency and lower titers compared to their respective unmodified wild-type AAV counterparts. Remarkably, these hybrid AAV constructs have exhibited novel attributes, such as the ability to be tailored for precise targeting of particular cancer cells and the capacity to interfere with immune responses by mitigating the generation of neutralizing antibodies and enhancing resistance to neutralization by Nabs [26–28].

Nonetheless, the modification of AAV capsid properties presents challenges that demand a thorough understanding of the essential virus components involved in recombinant virus production and entry interactions. Further investigation is necessary to comprehensively elucidate the precise chemistry of AAV capsids and their interactions with cells, thereby laying the foundation for enhanced and targeted gene therapies.

We have examined the potential for several specific residues within the AAV9 capsid to be replaced with azido-lysine, ensuring that it doesn't interfere with AAV production and maintains the ability to attach a Cy5.5 molecule to these altered residues. This assessment involved evaluating the infectivity of these modified versions of AAV9. From our investigation, we have identified five residues that can successfully be substituted with azido-lysine (AzK), resulting in titers comparable to the wild-type AAV9. However, these modified versions exhibit varying transduction capacities. Additionally, we have noticed that the attachment of the Cy5.5 molecule to the AAV9 capsid leads to differing outcomes, depending on the specific residue of attachment and the proportion of modified residues within the capsid.

## Results

### Discovery of specific AAV9 capsid residues amenable to replacement with azido-lysine while maintaining unaffected production of recombinant particles

**Fig. 1:**
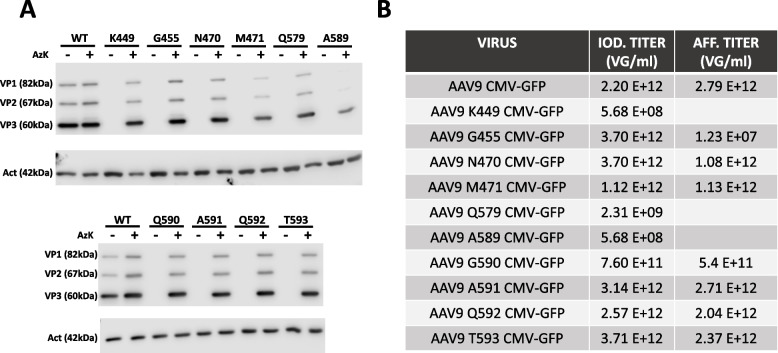
Analysis of AAV9 WT and mutant capsid proteins expression and AAV productions. **A** Western Blot analysis of VP1, VP2, and VP3 protein expression 72 hours post-transfection of AAV9 wild-type (WT) and mutated PDP9 plasmids together with the pAcBac1-tR4-MbPyl plasmid in the presence or absence of the unnatural amino acid AzK. **B** Presentation of AAV9 and mutant AAV titers obtained after the initial purification step using an iodixanol gradient, shown in the first column. The second column displays the titers obtained after the second purification step, which involved affinity chromatography in addition to the first purification step

Adeno-associated virus serotype 9 (AAV9) presents unique characteristics that make it a promising vector for delivering therapeutic genes into target cells. It exhibits a wide tropism for various tissues with a high transduction efficiency making it suitable for treating a range of genetic disorders that affect different parts of the body showing promising results in preclinical and clinical studies across a range of genetic disorders [29]. It has been used successfully in various animal models and has gained approval for treating spinal muscular atrophy in humans [30]. Taking these facts into consideration, we explored the possibility of using AAV9 as a platform to generate chimeric AAVs with enhanced properties, achieved by attaching functional moieties to specific residues of AAV9 capsid using straightforward click chemistry reactions. We aimed to replace specific residues with azido-lysine (AzK) within non-conserved regions of the AAV capsid. These regions encompassed residues that had been effectively modified in AAV2 or were pertinent to AAV9's galactose binding, as well as those situated in prominently exposed positions. Initially, we evaluated whether the replacement of the native residue with AzK impacted the stability of AAV9 capsid proteins. This was accomplished by transfecting 293 cells with the corresponding AAV production vectors containing the respective codons substituted with TAG, along with the necessary plasmids for introducing non-natural amino acids into the TAG codon, both in the presence and absence of AzK. All the replaced residues resulted in the production of the three capsid proteins in a manner similar to the wild-type version (Fig. 1A). Consequently, we proceeded to generate the 10 mutant recombinant AAV9 vectors. Notably, three of the mutant AAVs (K449, Q579, and A589) produced empty capsids following the iodixanol purification step, as it indicated the presence of the three capsid proteins while the AAV genomes were practically absent (Fig. 1B, Suppl Fig 1). Subsequently, we subjected the remaining seven mutants and the wild-type AAV9 to affinity purification to remove the majority of impurities present in the viral preparation post-iodixanol purification. This step aimed to obtain purer AAV preparations for the purpose of performing chemical modifications on the AAV capsid. Curiously, the mutant G455 could not undergo affinity purification (Fig. 1B). Notably, this specific residue has been identified as a critical position for the recognition of neutralizing antibodies [31]. It is plausible that the substitution of this residue prevents the binding of the antibody present in the affinity purification column. Out of the six purified mutants, the yield from five of them was comparable to that of the wild-type AAV9. However, one mutant, Q590, exhibited a lower titer even after the iodexanol purification step. This suggests that this particular AAV mutant was produced with lower efficiency. Hence, we chose the mutants N470, M471, A591, Q592, and T593 for chemical modification and subsequent characterization.

### Attachment of Cy5.5 to AAV9-AzK mutant versions

**Fig. 2:**
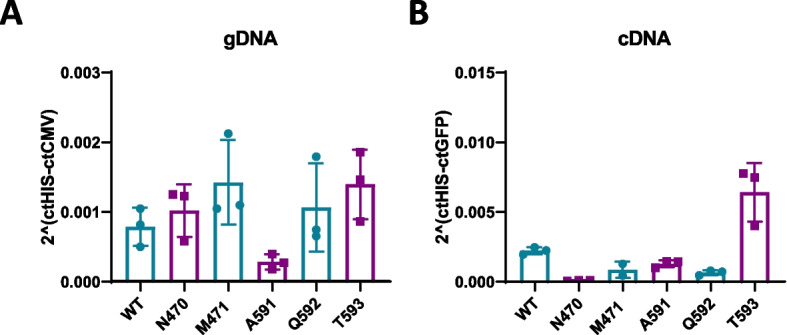
AAV9 and mutant AAV infectivity. Hela cells were exposed to AAV9 wild-type (WT) and mutant vectors at a multiplicity of infection (m.o.i.) of 2E5 vg/cell. After 72 hours of infection, the viral genome was quantified by assessing the presence of the CMV promoter (**A**), and expression levels were determined by measuring GFP transgene expression (**B**)

Prior to modifying the capsids of the AAV9 mutants, we evaluated the transduction capacity of each mutant by analyzing AAV genome and GFP mRNA expression in Hela cells 72 hours after vector administration (Fig. 2). Evaluation of AAV genomes presence showed that the five mutants presented a similar amount of AAV genomes as the wild type AAV9 at the end of the experiment. Nevertheless, upon analyzing GFP mRNA expression, it was evident that the N470 mutant exhibited no transgene expression. On the other hand, the T593 mutant demonstrated higher expression compared to the wild-type AAV9, while the remaining three mutants displayed slightly reduced expression levels. Consequently, we excluded the N470 mutant from the chemical modification experiments due to its lack of activity.

**Fig. 3:**
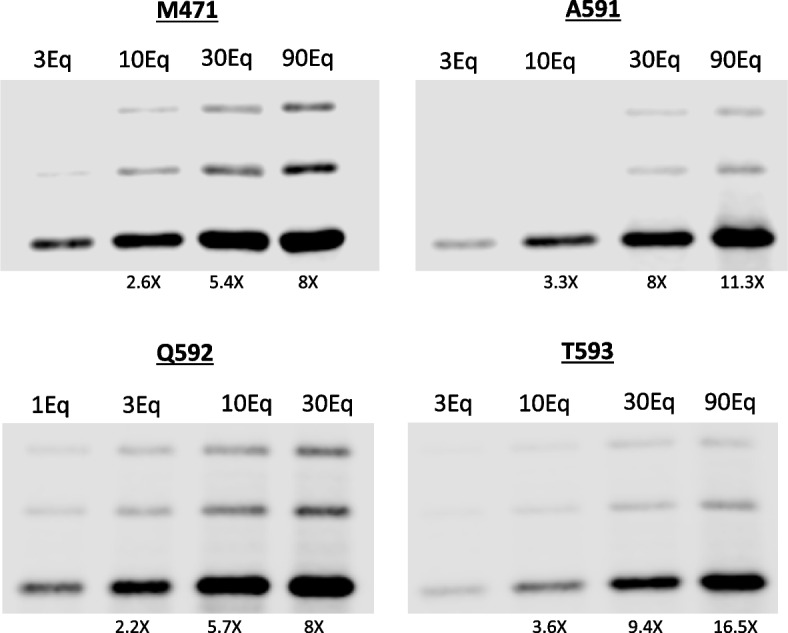
Attachment of Cy5.5 to AAV9 mutant versions. SDS-PAGE analysis of 2E10 VG of each mutant modified with different equivalents of CY5.5-DBCO molecules per azide group was performed. The resulting proteins were then assessed by measuring emitted fluorescence at a wavelength of 700 nm. The increment relative to the first lane is specified beneath each lane for every mutant

Next, we opted for Cy5.5-DBCO, a fluorescent molecule containing a DBCO group, to examine the accessibility of AzK residues for potential modification through a straightforward copper-free click chemistry reaction. Consequently, we subjected the various mutants to incubation with varying quantities of Cy5.5-DBCO equivalents at a temperature of 25ºC for a duration of 2 hours. Following this, we quantified the integrated fluorescent molecules by assessing the AAV capsid proteins via SDS-PAGE and subsequent visualization of the proteins using an Odyssey imaging system (Fig. 3). Among the AAV preparations, the M471 variant exhibits an increased ratio of empty capsids (Suppl Fig 2), leading to a relatively elevated initial fluorescent signal compared to the other mutants. Employing a greater quantity of Cy5.5-DBCO equivalents during incubation results in a heightened integration of the fluorescent molecule into the AAV capsids, demonstrating consistent enhancements across all mutant variants.

**Fig. 4:**
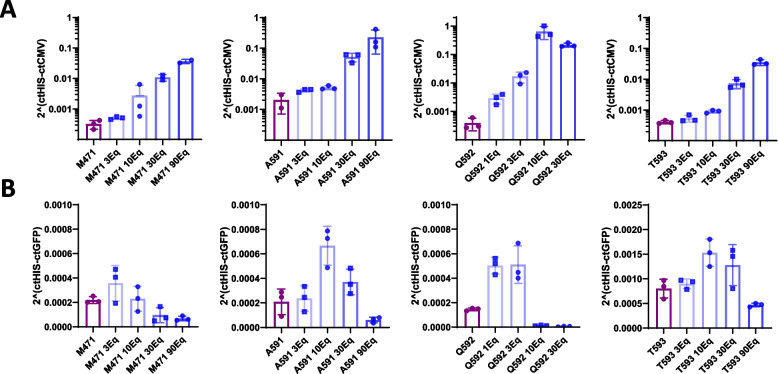
Modified AAVs infectivity. Mutant vectors modified with varying equivalents of CY5.5 were used to infect Hela cells at a m.o.i. of 2E5 vg/cell and analyze AAV genomes (**A**) and GFP transgene expression (**B**) 72 hours later

After establishing the conditions for similar Cy5.5 molecule attachment across the four positions on the AAV9 capsid, we proceeded to assess whether this capsid modification impacted the transduction efficiency of AAV in cell culture. Consequently, we inoculated Hela cells with equivalent quantities of each AAV9 mutant variant, both unmodified and modified using the four different proportions of Cy5.5-DBCO. Following a 72-hour incubation period, we analyzed the AAV genomes within the cells (Fig. 4A). Intriguingly, we noted a proportional increase in the quantity of AAV genomes present in the cells, aligning with the extent of AAV capsid modification. The AAVs linked with a higher concentration of Cy5.5 exhibited nearly 100 times more genomes than the unmodified mutant version. As a result, the modified AAV mutants demonstrate higher transgene expression compared to the unmodified versions (Fig. 4B). However, there is a threshold of modification beyond which a negative impact on transgene expression is observed. This negative effect results in lower expression than that achieved with the unmodified mutant, despite having nearly 100 times more genomes. Interestingly, the choice of the modified residue has a significant impact, ranging from a complete blockage of transgene expression (Q592) to an expression level similar to that of the unmodified mutant version (T593).

**Fig. 5:**
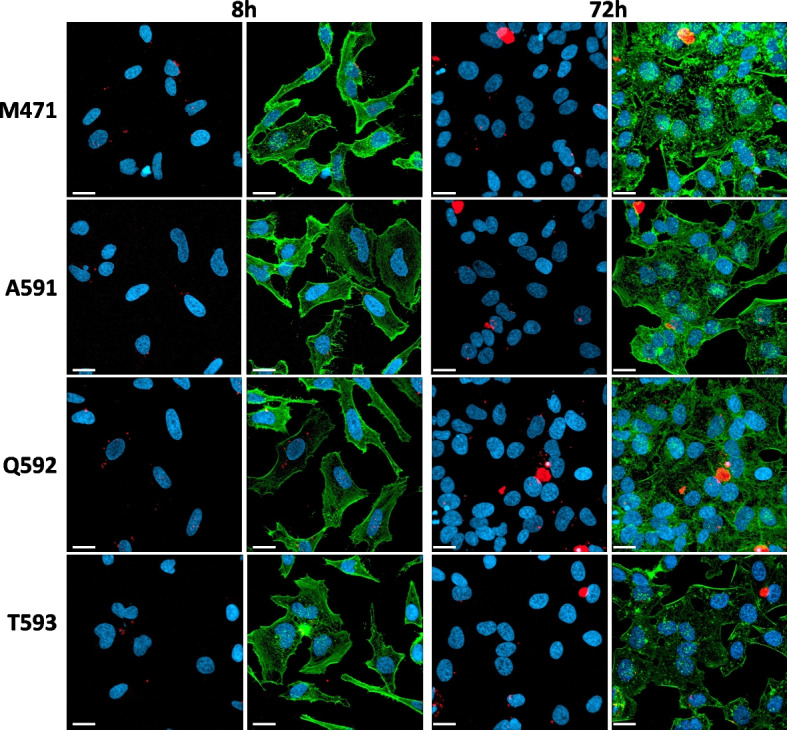
AAV Transduction Visualization. Hela cells were infected at a multiplicity of infection (m.o.i.) of 2E5 vg/cell, where mutants were chemically modified with 90 equivalents of Cy5.5, except for the Q592 mutant, which underwent modification with 30 equivalents. At eight and 72 hours post-infection, cells were fixed and prepared for visualization under a confocal microscope. Each image represents a typical view of each mutant. The actin cytoskeleton is marked in green, the nucleus in blue, and the CY5.5 in the capsids of the AAV mutants is observed in red. White bars correspond to 20 μm

Having a fluorescent molecule within the AAV capsid enabled us to track the localization of AAV particles in Hela cells at various time points following AAV infection. This allowed us to analyze how each mutant capsid behaves when the Cy5.5 molecule is attached to the viral particle. Initially, we assessed AAV infection after 8 hours of vector administration. At this point, we observed small spots ranging from 0.5 to 2 µm in size, indicative of AAV particles. Notably, these spots started to be detected when the M471 and Q592 mutants were subjected to the second-highest concentration of Cy5.5 in the reaction (30 Eq for M471 and 10 Eq for Q592), while the other two mutants just when were exposed to the highest concentration (Fig. 5, Suppl Fig 3). Furthermore, a significant number of these spots localized near the perinuclear region. At the end of the experiment, 72 hours after vector inoculation, we also observed the formation of big capsid aggregates in some of the infected cells, in addition to the smaller spots mentioned earlier (Fig. 5).

## Discussion

We have identified multiple residues within the AAV9 capsid that can be replaced with a reactive non-natural amino acid like AzK, without any adverse impact on rAAV production. In fact, we achieved comparable titers to those of the wild-type AAV9. Furthermore, our investigation revealed that the attachment of Cy5.5 to various positions on the AAV9 capsid exerts differential effects on AAV9's transduction capacity. Additionally, the quantity of molecules attached to the AAV9 surface also plays a role in influencing these effects.

Several authors have developed rAAVs incorporating reactive non-natural amino acids at various positions, employing different serotypes in their studies [26–28, 32]. These investigations have encountered challenges in generating mutated vectors with titers comparable to the wild-type version. Notably, AAV2 and AAVLK03 yielded significantly lower titers compared to AAV-DJ and AAV8, failing to surpass 15% of the wild-type vector titer in any of these cases. Conversely, within the AAV9 capsid, we successfully identified several positions where AzK substitution maintained production levels on par with wild-type AAV9. This finding highlights the necessity for a comprehensive screening process to pinpoint substitution positions that minimally impact rAAV production. Furthermore, it's important to consider that different types of unnatural amino acids can be incorporated into the AAV capsid, and the choice of the introduced reactive amino acid may influence the formation of recombinant particles.

After successfully producing reactive rAAVs at normal levels, the next step is to evaluate the infectivity of the generated vectors. We observed that four out of the five produced rAAVs displayed similar or slightly reduced transgene expression, whereas the substitution at position N470 led to a significant reduction in expression. Previous research has identified N470 residue as a crucial element in AAV9 binding to galactose before receptor attachment playing a transcendent role in AAV9 transduction [33]. Interestingly, there is no apparent correlation between the quantity of AAV genomes detected within the cells and the transgene expression levels in the mutants at positions N470, M471, and Q592, compared to those observed in AAV9 and the other two analyzed mutants. Similarly substitution of AAV2, AAV8 and AAV-DJ capsid residues showed variable effects on AAV transduction ranging from normal transduction to blockade [26–28, 32].

Introducing reactive residues into the AAV capsid enables the engineering of these vectors by attaching various moieties to the AAV surface, thereby imparting new properties. This approach has led to modifications in AAV tropism through the attachment of cyclic peptides [32], folic acid, or aptamers [27], and in some instances, it has even restored vector transduction capacity. Furthermore, site-specific pegylation and oligo conjugations have been employed to engineer viral shielding, providing protection to AAV particles against neutralizing antibodies [26, 28]. One of the primary features of these click-chemistry reactions at specific AAV capsid positions is the ability to incorporate chemical elements of interest at precise locations on the capsid. This stands in contrast to the unspecific chemical attachment of moieties that activate exposed amines on AAV capsids. Considerable effort has been dedicated to studying the impacts of attached molecule properties and positions. However, there has been limited characterization of the effects observed when varying proportions of a molecule are linked to the AAV capsid. We have noticed that the incorporation of an inert molecule such as Cy5.5 has varying effects on AAV transduction, depending on both the proportion of attached molecules and the specific residue used for attachment. Interestingly, when the rAAV carries a higher proportion of attached molecules on the AAV capsid at all four assessed positions, there is a greater presence of AAV genomes within the cells. This increase is linked to enhanced transgene expression. However, a blockade of transgene expression occurs when more Cy5.5 molecules are attached to these four positions, with the magnitude of this effect varying depending on the modified residue. Hence, the proportion and location of attached molecules on the AAV capsid can influence the intended outcomes when creating chimeric AAVs. Numerous studies have examined the biological effects associated with molecule placement at various positions. These studies have revealed the challenges in predicting the biological impact based solely on the selected modified residue. Consequently, it is essential to experimentally identify the specific attachment points for each AAV serotype. For instance, when folic acid is attached to the AAV DJR/A-N589 residue, it enhances AAV transduction in tumor cells. In contrast, when folic acid is attached to AAV LK03-N588, it completely abolishes viral transduction [27].

However, a comprehensive exploration of the effects stemming from different proportions of attached molecules has been lacking. Most of these studies have not provided a detailed characterization of the molecule density on the AAV capsid. Interestingly, distinct outcomes are achieved when GalNac or Mannose is covalently linked to the AAV2 surface through the formation of a thiourea bond with the accessible amino groups or tyrosine bioconjugation of the rAAV capsid [23, 34, 35]. While there is no discernible effect on AAV2 transduction efficiency when ten times more Mannose is attached, an equivalent increase in GalNac leads to a notable reduction in AAV2's infectivity in the target organ. Likewise, the elevation of Cy5.5 density on the AAV9 surface exerts a detrimental effect on AAV transgene expression in all four positions examined in our study. However, this effect coincides with an increase in the presence of AAV genomes within the cells. This suggests that the deficiency in transgene expression is not linked to AAV entry but rather to the localization of the AAV genome. Furthermore, 72 hours after AAV administration, we observed an accumulation of AAV9 capsids in the cells in all four modified mutants. These capsids may contain AAV genomes, which, if present, would be inactive as they require access to the cellular nucleus to become transcriptionally active. In contrast, the attachment of Mannose or GalNac to AAV2 and AAV8 capsids enhances transgene expression, normalizing or even improving transgene expression in the liver and retina, despite a significant reduction in AAV genomes [23, 34]. We need to investigate whether attaching these glycans to the AAV9 surface at any of the four studied residues can enhance transgene expression in the target organs, similar to what has been observed in AAV2 and AAV8.

## Materials and methods

### Cloning and construction of rAAV vector

Mutant Cap9 versions were generated using the In-Fusion® cloning system (Takara) using as template the pAAV-MCS-AAV9-Cap-SalI vector obtained subcloning the Sal I DNA fragment containing the Cap9 gene obtained from the pDP9 vector into the pAAV-MCS plasmid to facilitate nucleotide substitutions. The cloning reactions were performed according to manufacturer indications. To generate each mutation, there are required two PCR products that were performed using the appropriate primers presented in Table 1. The forward primer for each mutation was paired with the SapI-AAV9 CAP-R primer, while the reverse primer for the mutation was coupled with the XcmI-AAV9 CAP-F primer. To introduce the mutations into the pAAV-MCS-AAV9-Cap-Sal I vector it was digested with Sap I and Xcm I and the obtained 6.6 kb DNA was used in the recombination ligation with the corresponding pair of PCR products. Next, the Cap9 gene with desired mutation was introduced into the pDP9 vector using Sal I restriction enzyme to transfer a 3 kb DNA fragment with the mutated gene into the host vector pDP9 using regular cloning methods. All plasmids were characterized by restriction analysis and sequencing.

**Table 1:** Primers

**PRIMER**	**SEQUENCE**
K449-AAV9 CAP-F	5’ TTAATAGTCTATGAGAGATAGTACAAGTATT 3’
K449-AAV9 CAP-R	5’ TATCTCTCATAGACTATTAACGGTTCTGGACA 3’
G455-AAV9 CAP-F	5’ GATTCTGCTAAGAACCGTTAATAGTCTTTG 3’
G455-AAV9 CAP-R	5’ TAACGGTTCTTAGCAGAATCAACAAACGCTAAA 3’
N470-AAV9CAP-F	5’AGCCATCTAGCTGGGTCCGGCCACACTGAA 3’
N470-AAV9CAP-R	5’ CCCAGCTAGATGGCTGTCCAGGGAAGAAAC 3’
M471-AAV9CAP-F	5’ GACAGCCTAGTTGCTGGGTCCGGCCACACT 3’
M471-AAV9CAP-R	5’ AGCAACTAGGCTGTCCAGGGAAGAAACTAC 3’
Q579-AAV9CAP-F	5’ TTTGTGGCCACCTATCCATAGGACTCCGTTGCTA 3’
Q579-AAV9CAP-R	5’ TATGGATAGGTGGCCACAAACCACCAGAGT 3’
A589-AAV9CAP-F	5’ CGCCTGCTATTGGGCACTCTGGTGGTTTGT 3’
A589-AAV9CAP-R	5’ GCCCAATAGCAGGCGCAGACCGGCTGGGTT 3’
Q590-AAV9CAP-F	5’ CTGCGCCTATGCTTGGGCACTCTGGTGGTT 3’
Q590-AAV9CAP-R	5’ CAAGCATAGGCGCAGACCGGCTGGGTTCAA 3’
A591-AAV9CAP-F	5’ GGTCTGCTACTGTGCTTGGGCACTCTGGTG 3’
A591-AAV9CAP-R	5’ GCACAGTAGCAGACCGGCTGGGTTCAAAAC 3’
Q592-AAV9CAP-F	5’ GCCGGTCTACGCCTGTGCTTGGGCACTCTG 3’
Q592-AAV9CAP-R	5’ CAGGCGTAGACCGGCTGGGTTCAAAACCAA 3’
T593-AAV9CAP-F	5’ CCAGCCCTACTGCGCCTGTGCTTGGGCACT 3’
T593-AAV9CAP-R	5’ GCGCAGTAGGGCTGGGTTCAAAACCAAGGA 3’
XcmI-AAV9 CAP-F	5’ CACGCTGACTTGGCCAGTAGAATACTGGGT 3’
SapI-AAV9 CAP-R	5’ CAACTTCAAGCTCTTCAACATTCAGGTCAA 3’

### Cell transfection

Human embrionic kidney cells (HEK 293, ATCC^®^ CRL-3216^™^) were grown in Dulbecco's modified Eagle's medium with pyruvate (DMEM 11995073, GIBCO) supplemented with 10% heat-inactivated fetal bovine serum (FBS 10270-106, GIBCO), 1% penicillin and streptomycin (15140-122, GIBCO). One day before transfection, 5x10^5^ HEK 293 cells were plated in M6-wells. The next day, medium was replaced with 1800 μL of DMEM FBS 2% medium and 200 μl of a previously prepared transfection mix containing 3 μg of pDP9 plasmid (wild-type or mutant versions), 2 μg of pAcBac1.tR4-MbPyl plasmid (Addgene, #50832) and 11.25 μL of PEI 10 mM (Polyethylenimine, Sigma Cat# 40872-7). To obtain mutated proteins expression 20 μL of amino acid H-L-Lys (EO-N3)-OH*HCl 100 mM (AzK, Iris) were added 1 hour and 24 hours after transfection. Transfections were incubated for 72 hours.

### Protein analysis

Protein extracts were obtained from cell pellets resuspended in resuspension buffer (Tris 0.5 M pH 7.4, SDS 10%, H2O miliQ and a mix of proteases inhibitors composed of PMSF (Phenylmethylsulfonyl fluoride), Aprotinine, Sodium orthovanadate and Pyrophosphate). Next, the same volume of Loading Buffer 2X (Bio-Rad Cat# 1610737) supplemented with β-mercaptoethanol at 5% was added and cell extracts were analyzed by SDS-PAGE. Finally proteins were transferred to nitrocellulose membrane for protein immunodetection using the corresponding antibodies to detect AAV capsid proteins (Origene [BM5051]) and actin (Abcam) and the *Lumigen ECL ULTRA substrate A+B* (Lumigen). Then, membranes were scanned with the *Oddyssey® Fc Imaging System* (Li-Cor) selecting the 700 nm channel for 30 seconds and the chemiluminiscence exposition for 2 minutes.

To visualize capsid viral proteins present in the purified AAV preparations and after chemical modification samples were analyzed in a 7.5 % SDS-PAGE. Once the electrophoresis had ended, the gel was fixed (10% ethanol, 7% glacial acetic acid) for 30 minutes and next gel was incubated with SYPRO Ruby protein stain (BioRad) for 16-18 hours and it was visualized with the Quantity ONE program (BioRad). Cy5.5 incorporation was observed after gel fixation at the *Oddyssey® Fc Imaging System* (Li-Cor).

### Production of rAAV vectors

The viral particles were produced by polyethyleneimine-mediated co-transfection in HEK-293T cells in 150 mm plates until nearly confluent. Nearly confluent cells were co-transfected with three different plasmids - the helper/packaging plasmid (pDP9 wild type or mutated), the AcBac1.tR4-MbPyl plasmid which contain the genes that encodes pyrrolisyl-tRNA synthetase and its corresponding tRNA necessaries to introduce the desired non-natural amino acid into the amber mutation and the ssAAV-CMV-GFP plasmid - using linear polyethyleneimine 25 kDa (Polysciences, Warrington, PA). One hour after DNA transfection, 180 μL of the amino acid H-L-Lys (EO-N3)-OH*HCl 100 mM (AzK) were added to the medium and 90 μL of it 24 hours later to produce the mutant AAV9 versions. Transfected cells were cultured for 72 hours at 37ºC 5% CO2 before being harvested for AAV purification. Cell culture supernatant was collected and treated with polyethylene glycol solution (PEG8000, 8% v/v final concentration) for 48–72 h at 4 °C. It was then centrifuged at 1400×g for 15 min, the pellet was resuspended in lysis buffer (50 mM Tris-Cl, 150 mM NaCl, 2 mM MgCl_2_, 0.1% Triton X-100) and kept at −80 °C. After three cycles of freezing and thawing, VPs obtained from cell supernatants and lysates were purified by ultracentrifugation at 350,000×g during 2.5 h in an iodixanol (Optiprep 415468 ATOM) gradient. Once our viral particles were purified by iodixanol gradient ultracentrifugation those samples were purified by affinity chromatography. For this step the ÄKTA start + Frac 30 + UNICORN 1 control system from ÄKTA purification systems (GE Healthcare Life Sciences) and the POROS™ GoPure™ AAVX pre-packed 1mL columns (Thermo Fisher Scientific) were used. Our samples were diluted, in the equilibration buffer (PBS, pH 7.4) until a concentration of 5e11vg/ml was obtained and a 0.0001% of Pluronic acid was added to this solution. A method for the purification of the viral particles was created using the UNICORN 1 software. After the method was run, fractions containing the purified viral particles were mixed and subjected concentration and buffer exchange with PBS 5% sucrose with 0.001% of Pluronic acid, using an Amicon Ultra centrifugal device with Ultracel 100K (UFC910008 Millipore) in a total volume of 2 ml. to the same procedure as described above to exchange buffer with PBS 5% sucrose.

Titration of VP was performed by qPCR using primers complementary to the CMV region: 5´-TTACCATGGTGATGCGGTT-3´ and 5´-TACACGCCTACCGCCCATT-3´. Viral genomes were extracted from DNAase-treated viral particles using the High Pure Viral Nucleic Acid Kit (Roche).

### Chemical conjugation of Cy5.5 to the AAV9 capsid

The attachment of Cy5.5 to the AAV capsid relied on click chemistry reactions involving the DBCO group present in CY5.5 and the azide groups present on the mutant capsids. Each reaction maintained a virus concentration of 1.33E9 viral genomes (VG) per 1 µl of reaction. The reaction included an excess of CY5.5-DBCO equivalents based on the azide groups present. To determine the azide quantity, we considered that each capsid comprises 60 monomers, each containing one azide group. Additionally, we assumed a 10:1 ratio of empty to full capsids, a parameter used for calculating infectious particle quantities.

Reactions were conducted in PBS 5% Sucrose, 0.0001% pluronic acid, with agitation at 300 rpm for 2 hours at a temperature of 23ºC, utilizing 1.5 ml Eppendorf tubes with no more than 1 ml of reaction volume.

The modified virus was purified through filtration using Amicon units (Amicon Ultra-0.5 Centrifugal Filter Unit – Sigma (UFC501096)). This process eliminated unreacted molecules, leaving the virus in a medium of 5% sucrose PBS, 0.0001% pluronic acid. A 500 μl portion of the reaction mixture was loaded onto a 0.5 ml Amicon and centrifuged at 14000 g for 10 minutes at 4ºC. This step was repeated with an additional 500 μl of sucrose PBS. The purified virus was obtained by inverting the Amicon into a clean tube and centrifuging at 1000 g for 2 minutes at 4ºC.

### Cell infection and molecular analysis

200000 Hela cells were seeded in M6 wells and next day they were infected with a m.o.i. of 2E5 vg/cell with the corresponding AAV vectors. 72 hours later they were harvested for molecular analysis or for immunofluorescence visualization of the AAV particles.

Vector genome copies present in cells were determined by qPCR using iQ™ SYBR® Green (BioRad) in a CFX96 Real-Time Detection System (BioRad) with primers specific for the CMV promoter (F 5’ TTACCATGGTGATGCGGTT; R 5’ TACACGCCTACCGCCCATT). Mouse *Gapdh* (glyceraldehyde-3-phosphate dehydrogenase) (F 5’ TGCACCACCAACTGCTTA R 5’ GGATGCAGGGATGATGTTC) was used as housekeeping gene.

To analyze transgene expression, total RNA was isolated from cell using the Maxwell® 16 LEV simplyRNA Tissue Kit (Promega) according to the manufacturer’s instructions and quantified. Extracted RNA was reverse transcribed into complementary DNA (cDNA) using M-MLV reverse-transcriptase (Invitrogen). Copies of GFP (F 5’ ATGGTGAGCAAGGGCGAGGA; R 5’ TTGCCGGTGGTGCAGATGAA) cDNA were determined by qPCR using GoTaq qPCR Mastermix (Promega) in a CFX96 Real-Time Detection System (BioRad). Mouse histone expression levels were used for normalization (F 5’ AAAGCCGCTCGCAAGAGTGCG; R 5’ ACTTGCCTCCTGCAAAGCAC).

### Immunofluorescence

For the stress fibers staining, HeLa cells were fixed with 4% paraformaldehyde, permeabilized with 0.1% Triton X-100 and incubated with Oregon Green® 448 phalloidin (Invitrogen) for 15 min at 37 °C. The stained cells for each preparation were mounted with Vectashield Mounting Medium with DAPI (Vector Laboratories). The samples were analyzed using the 40x objective of an Axiovert 200M confocal LSM 510 META Zeiss microscope.

### Statistical analysis

Data are presented as mean values ± SEM and were statistically analyzed using a one-way ANOVA or Kruskal-Wallis test, Bonferroni multiple comparison test, two-way ANOVA test for weight analysis, or unpaired *t* test when only two groups were compared with GraphPad Prism 9.05 software (GraphPad Software Inc., CA, USA). *P* < 0.05 was considered significant.

### Supplementary Information

Supplementary Material 1.

## Data Availability

No datasets were generated or analysed during the current study.

## Abbreviations

AAV: Adeno-associated virus
EMA: European Medicines Agency
FDA: Food and Drug Administration
Nabs: Neutralizing antibodies
AAVR: AAV receptor
VP: Viral Protein
GalNac: N-Acetyl-D-galactosamine
tRNA: Transfer RNA
AzK: Azido-lysine
DBCO: Dibenzocyclooctyne
Cy5.5: Cyanine5.5
GFP: Green Fluorescent Protein
mRNA: Messenger RNA
SDS-PAGE: Sodium dodecyl sulfate polyacrylamide gel electrophoresis
PCR: Polymerase Chain Reaction
Kb: Kilobase
