# Supplementary material for: The infectivity of AAV9 is influenced by the specific location and extent of chemically modified capsid residues
Source: J Biol Eng. 2024 May 14;18:34. doi: 10.1186/s13036-024-00430-7 (PMC11092203; doi:10.1186/s13036-024-00430-7)
Supplement: Supplementary file 1 — Supplementary Material 1. [file 13036_2024_430_MOESM1_ESM.zip › SUPPLEMENTARY FIGURE LEGENDS.docx]

**SUPPLEMENTARY FIGURE LEGENDS**

**Suppl. Figure 1. Analysis of AAV9 mutant capsid proteins presence in AAV preparations**. AAV9 mutants, including K449, G455, Q579, and A589, were produced and purified using an iodixanol gradient. Subsequently, SDS-PAGE analysis was performed, and proteins were visualized after SyPro staining.

**Suppl. Figure 2. Analysis of AAV9 WT and mutant capsid proteins presence in AAV purified preparations**. AAV9 wild-type (WT) and mutants (N470, M471, A591, and T593) productions underwent purification through iodixanol gradient and affinity chromatography. Subsequently, SDS-PAGE analysis was conducted, and proteins were visualized after SyPro staining.

**Suppl. Figure 3. AAV Transduction Visualization.** Hela cells were infected with all mutants, modified with either 30 or 90 equivalents of Cy5.5 except for the Q592 mutant, which underwent modification with 10 or 30 equivalents, at a multiplicity of infection (m.o.i.) of 2E5 vg/cell. Eight hours post-infection, cells were fixed and prepared for visualization under a confocal microscope. Each image corresponds to one mutant, with the actin cytoskeleton labeled in green, the nucleus in blue, and the CY5.5 in the capsids of the AAV mutants visualized in red. White bars correspond to 20 μm.
