# Supplementary figures and images for: The infectivity of AAV9 is influenced by the specific location and extent of chemically modified capsid residues
Source: J Biol Eng. 2024 May 14;18:34. doi: 10.1186/s13036-024-00430-7 (PMC11092203; doi:10.1186/s13036-024-00430-7)

## Slide 1
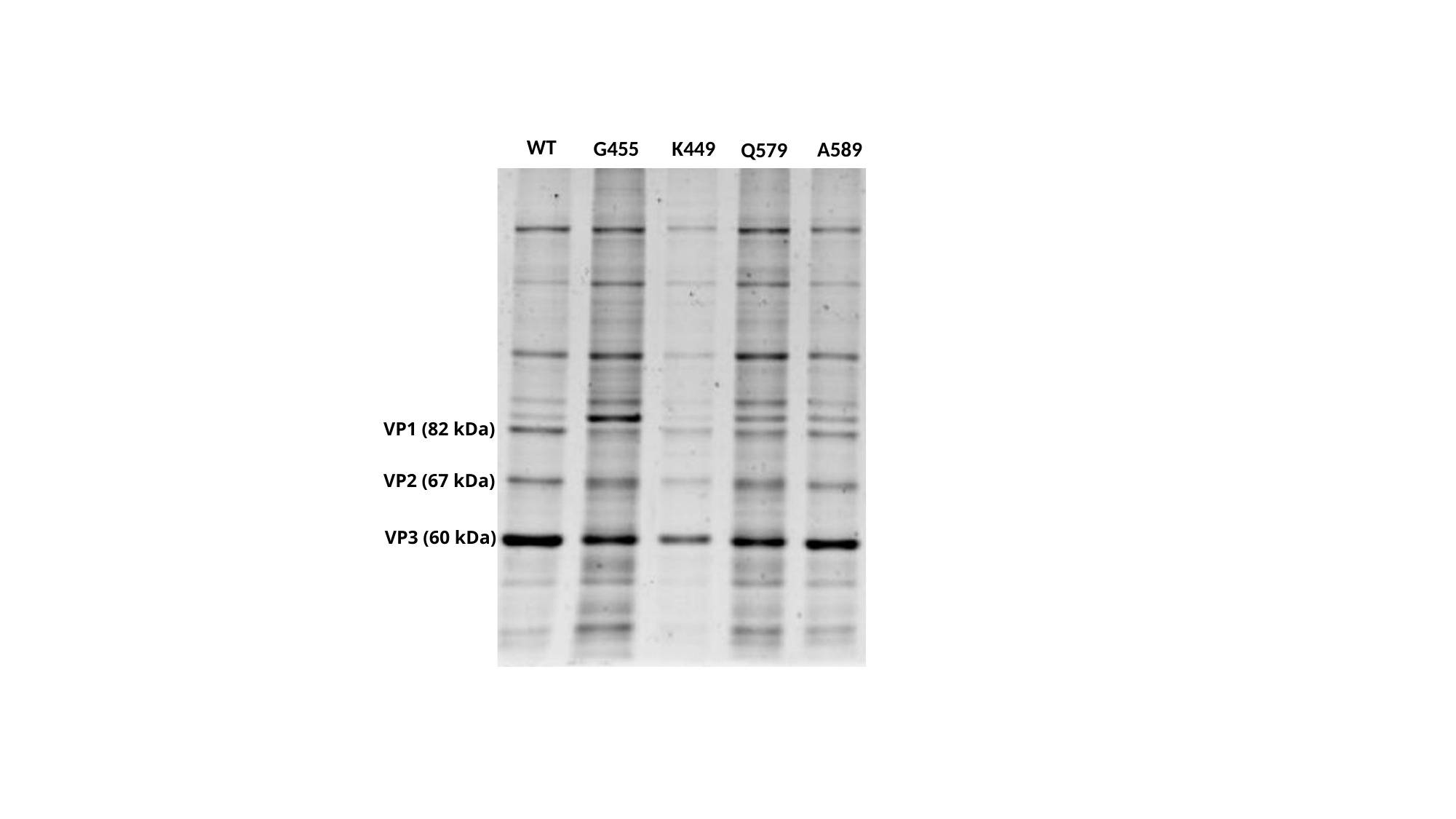

WT
K449
G455
A589
Q579
VP1 (82 kDa)
VP2 (67 kDa)
VP3 (60 kDa)

Supplement: Supplementary file 1 — Supplementary Material 1. [file 13036_2024_430_MOESM1_ESM.zip › Suppl Figure 1.pptx]

## Slide 1
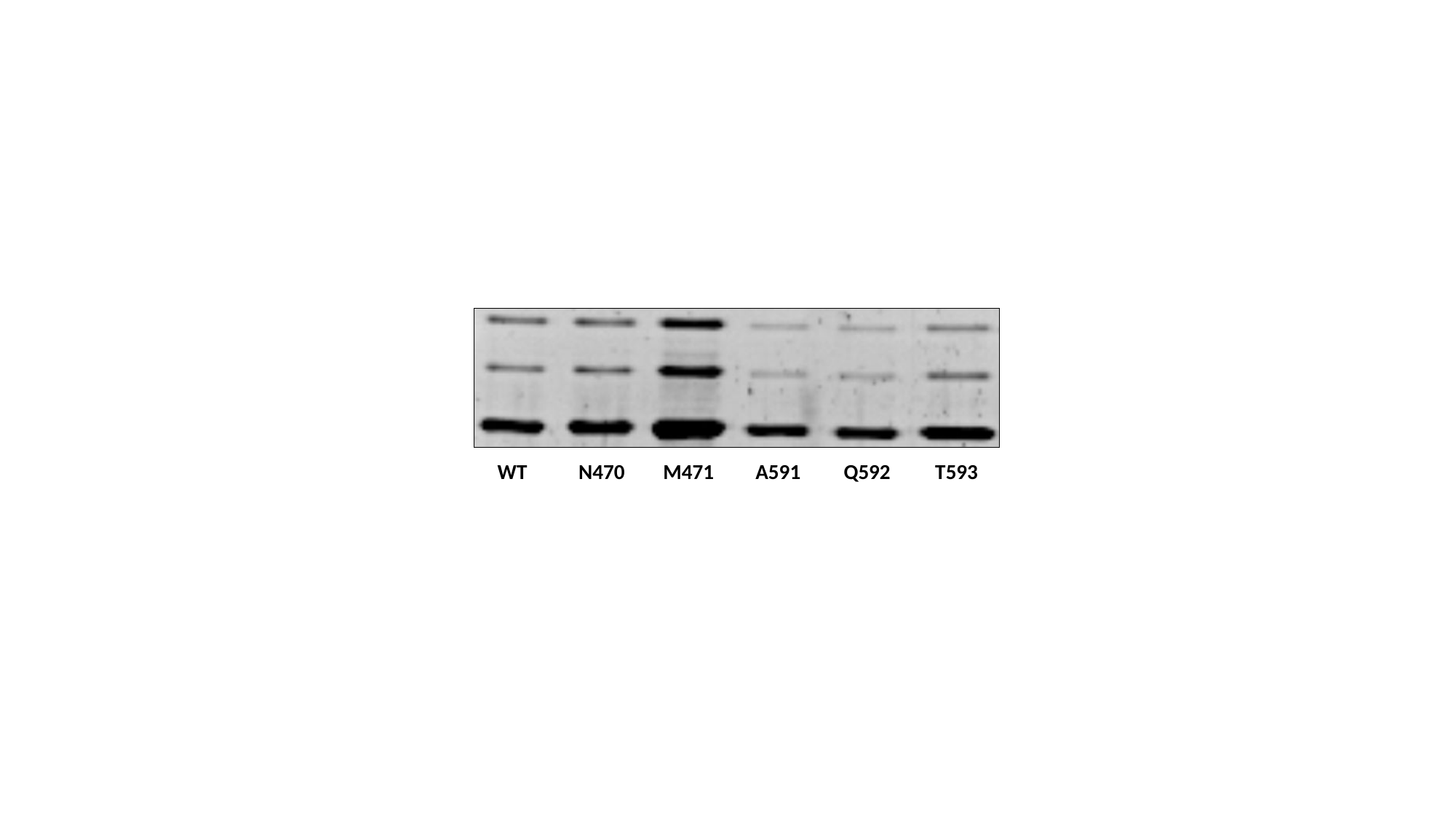

WT
N470
M471
A591
Q592
T593

Supplement: Supplementary file 1 — Supplementary Material 1. [file 13036_2024_430_MOESM1_ESM.zip › Suppl Figure 2.pptx]

## Slide 1
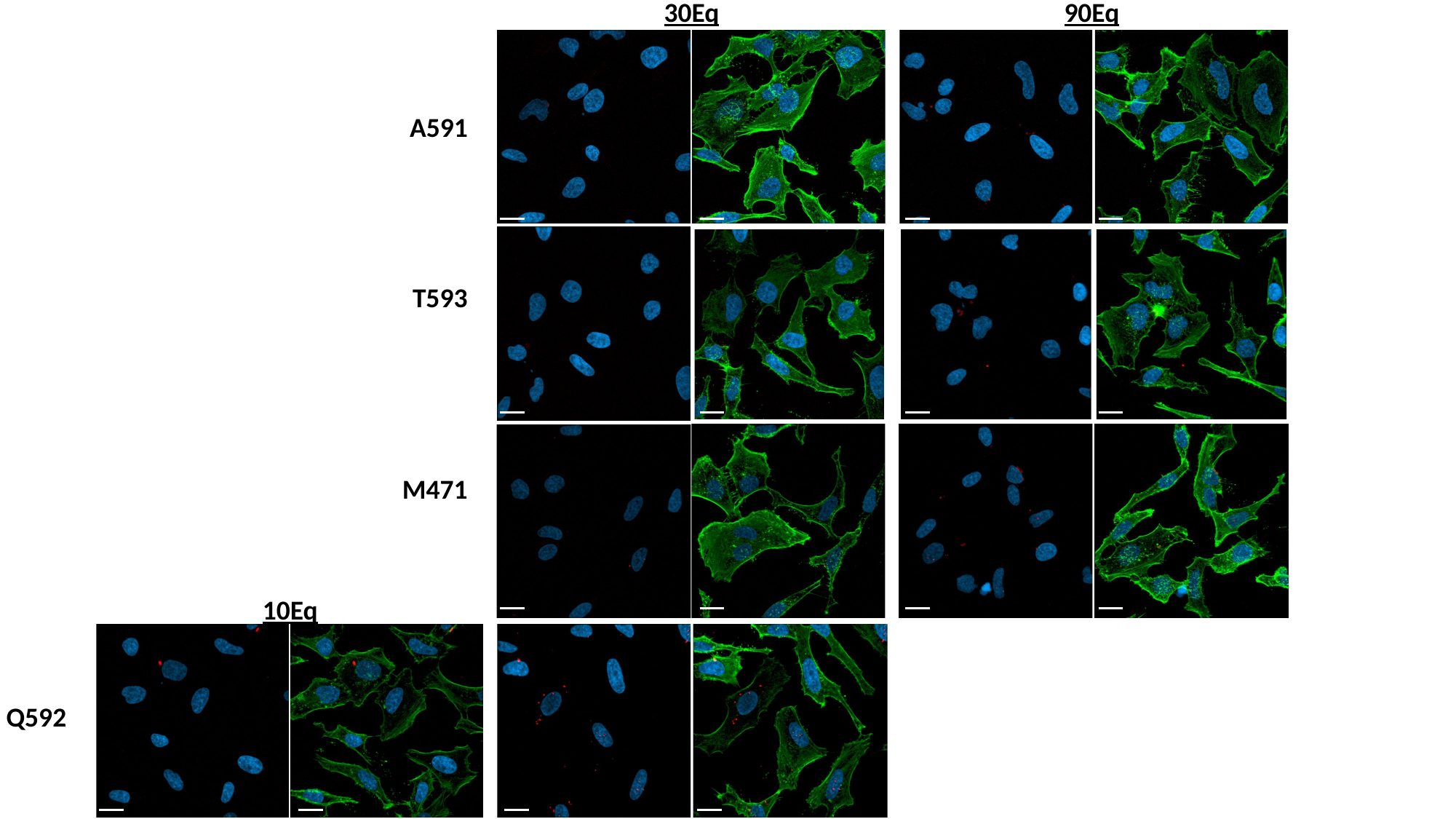

30Eq
90Eq
A591
T593
M471
10Eq
Q592

Supplement: Supplementary file 1 — Supplementary Material 1. [file 13036_2024_430_MOESM1_ESM.zip › Suppl Figure 3.pptx]
